# The research on female partners of injecting drug users-gender aspect and risk of HIV

**DOI:** 10.1186/1742-4690-9-S1-P96

**Published:** 2012-05-25

**Authors:** Miljana Grbic, Verica Lela Ilic, Sladjana Baros, Vesna Ciprus, Jelena Tadzic, Rade Grbic, Gordana Jurican, Milan Parlic, Svetomir Samardzic

**Affiliations:** 1 Unaids Focal Point for Serbia at Undp, Belgrade, Serbia

## 

The goal of this research is to assess whether there is an increased risk of HIV infection based on gender among female partners of IDUs and to what degree is this risk present. The research has been conducted using quantitative and qualitative methodology. For the purpose of collecting the research data among the female partners of IDUs and male partners who are IDUs, a qualitative methodology was used (in-depth interviews) complemented with a quantitative methodology (survey). Within the research 50 male injecting drug users (IDUs) have been reached and 50 steady female partners of the injecting drug users, regardless of the fact whether they themselves use drugs or not. Research findings indicate the following: Gender aspect is very important in initiation into drug use. Girls and women often start with drug use practice with their partner. Out of 19 IDU males who are in a relationship with a female partner who is an IDU, eight male subjects reported that their female partners had the first contact with drugs through them. On the other hand, out of 50 male participants, only one had the first experience with drugs during a relationship with a woman IDU. Sharing of injecting equipment is still present . In most cases a male partner has priority in distribution and taking drugs. Traditional understanding and acceptance of gender roles is reflected in attempts to explain or “justify” a man’s violent behaviour with the withdrawal crises or need for drugs. Sex work is one of the frequent activities for procuring money, or sex services are offered to drug dealers in exchange for drugs. The decision on purchasing and using a condom is mostly left to a male partner, and women and girls are not enough empowered to impose the use of a condom. Men are more prone to changing partners. Women prove their devotion to a partner by agreeing to risky behaviour (for ex. intercourse without a condom) or even by willingness to get infected with HIV. They tend to remain in a relationship with a person who is HIV positive, even when that person has infected them. In conclusion, the research findings have to a great degree confirmed gender specific risks for women that have been stated in the professional literature: unequal power relations and often weaker economic position of a woman. Women are in a weaker position when they make a decision about when, with whom and under what conditions they would have a sexual intercourse. The research findings, although they are not representative due to the size and sampling method, are certainly indicative – confirm to a great degree the thesis about the specific vulnerability of women and girls, both related to situations that lead to HIV infection, and related to availability and specific content of services offered to women living with HIV.

